# Assessing Patient Awareness of Sun Exposure and Eyelid Cancer

**DOI:** 10.7759/cureus.108315

**Published:** 2026-05-05

**Authors:** Carolyn May, Samantha Prabakaran, Alexis Cherry, Nikisha Richards

**Affiliations:** 1 Ophthalmology, Virginia Commonwealth University, Richmond, USA; 2 Ophthalmology, Virginia Commonwealth University School of Medicine, Richmond, USA

**Keywords:** cancer prevention, eyelid cancer, eyelid surgery, sun exposure, sun protection

## Abstract

Background and objective

Eyelid cancers are associated with significant diagnostic and therapeutic challenges. The eyelids are highly sun-exposed, yet awareness of eyelid cancer risk and sun protection practices remains insufficiently studied. This study aimed to evaluate patient awareness regarding eyelid cancer and associated preventive behaviors across different demographic groups.

Methods

A cross-sectional survey study was conducted at Virginia Commonwealth University (VCU) Health between May and August 2023. Ophthalmology clinic patients were given an anonymous 22-question survey upon their arrival. Demographics, awareness of sun exposure and eyelid cancer, and preventive practices were analyzed using the Chi-square tests of independence.

Results

A total of 484 participants completed the survey. While 191 of 197 White respondents (96.9%) recognized sun exposure as a risk factor for skin cancer, only 153 of 213 Black respondents (71.8%) did so (p < 0.0001). Overall, 214 of 484 participants (44.2%) were unaware that the eyelids can be affected by cancer. Only 70 of 484 respondents (14.8%) reported using facial sunscreen daily, and 115 of 484 (24.4%) reported applying sunscreen to the eyelids. Individuals with a higher level of education reported greater use of sun exposure preventative measures.

Conclusions

Awareness of eyelid cancer and adherence to sun protection practices remain suboptimal, particularly among Black patients, men, older adults, and those with lower levels of education. These findings highlight the need for targeted public health initiatives and physician counseling to improve prevention and reduce the risk of eyelid cancer.

## Introduction

Eyelid cancers account for approximately 5-10% of all skin malignancies, and the incidence in the United States is 15.7 cases per 100,000 people per year [1,2]. Basal cell carcinoma is the most common eyelid cancer in the United States, followed by squamous cell carcinoma [3,4]. Compared with other skin malignancies, eyelid cancers pose significant challenges due to the functional and aesthetic implications of treatment involving excision of eyelid skin and underlying structures [5]. Eyelid cancers can also be diagnostically challenging for family physicians, dermatologists, and ophthalmologists, as differentiating benign from malignant lesions in the periocular region can be difficult [6,7]. Risk factors associated with a higher risk of eyelid cancer include smoking, advanced age, male sex, White race, and increased UV exposure [3].

The eyelids are among the most sun-exposed areas of the skin. A lack of awareness regarding proper sun protection has been attributed to advanced presentations and poor prognoses in skin cancer cases [8]. In a 2005 National Health Interview Survey study involving 28,235 adult participants, more skin cancer risk behaviors were found in men, non-Hispanic Whites, individuals with lower levels of education, and younger individuals within certain age-stratified groups [9]. Previous survey-based studies have shown lower perceived skin cancer risk among African Americans, with 46% reporting zero skin cancer risk and 76% reporting zero or low risk [10]. Furthermore, 65% of participants from ethnic groups (African American, Asian, or Hispanic) believed that they were not at risk of skin cancer [11]. 

Suggested strategies for prevention of eyelid cancer include seeking shade during peak sun hours, wearing protective clothing including hats and sunglasses, applying sunscreen with a sun protection factor (SPF) greater than 30 to the face and eyelids, and avoiding the use of tanning beds [12,13]. Limited studies have addressed patient awareness of eyelid cancer risk and the use of preventive measures. In light of this, the present survey-based study aims to evaluate patient awareness of eyelid cancer and sun exposure across demographic groups to assist primary care providers, dermatologists, and ophthalmologists in providing informed guidance on sun protection methods and eyelid cancer prevention strategies.

## Materials and methods

Ethical approval

This study was approved by the Institutional Review Board of Virginia Commonwealth University (VCU) (IRB number: HM20025746) and adhered to the ethical principles outlined in the Declaration of Helsinki. All data collection conformed to the requirements of the U.S. Health Insurance Portability and Accountability Act of 1996 (HIPAA).

Study design and setting

A cross-sectional survey study was conducted at the VCU Health Department of Ophthalmology outpatient clinics in Richmond, Virginia, between May and August 2023. The study was anonymous and aimed to assess patient awareness of sun exposure risks and preventive behaviors related to skin and eyelid cancer.

Study population and eligibility criteria

All adult patients (≥18 years old) presenting for ophthalmology appointments during the study period were eligible to participate. Patients from the Department of Corrections and individuals younger than 18 years were excluded from participation. 

Sampling technique and data collection

A convenience sampling approach was used. Upon arrival at the clinic, patients were informed that the study was voluntary and anonymous and were offered the option to complete a 22-item multiple-choice survey while waiting for their appointments. The survey included questions on demographic information, including age, sex, race, and education level, as well as knowledge of risk factors for skin and eyelid cancer and behaviors related to sun protection and reduction of sun exposure.

Variables and potential confounders

The primary variables analyzed were awareness of skin cancer risk and adoption of preventive measures through survey scoring. Demographic characteristics, including age, gender, race, and education level, were assessed as potential confounding factors influencing awareness and preventive behavior.

Statistical analysis

Survey responses were analyzed using descriptive statistics to summarize demographic characteristics and response frequencies. Education level was categorized into three groups: (1) some high school, General Educational Development (GED), or completion of high school; (2) some college, associate degree, or bachelor’s degree; and (3) master’s or doctorate. Chi-squared tests of independence were used to evaluate associations between demographic characteristics and both awareness and preventive behaviors. Statistical significance was defined at a p-value < 0.05. Analyses were performed using Microsoft Excel (Microsoft Corporation, Redmond, WA).

## Results

Patient characteristics 

**Figure 1:**
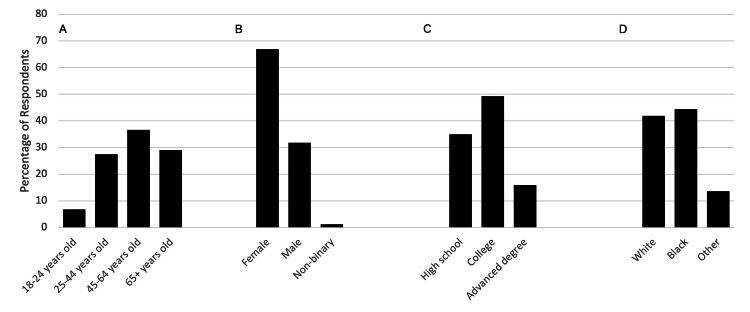
Demographic characteristics of survey respondents Panel A represents the age of respondents; panel B represents the gender of respondents; panel C represents the educational level of respondents; and panel D represents the race of respondents. Education level (C) is represented as “High school”, including respondents with some high school education, high school completion, or GED; “College”, including respondents with some college, associate degree, or bachelor’s degree; and “Advanced degree”, including respondents with a master’s or doctorate degree GED: General Educational Development

In total, 484 patients completed and returned the questionnaire. Demographic characteristics of survey respondents are summarized in Figure 1.

Awareness 

Table 1 summarizes the results of the chi-squared test of independence of patients’ awareness of sun exposure and skin and eyelid cancer across racial groups. Significant differences in awareness were observed, with Black participants exhibiting lower levels of awareness compared to other racial demographics.

**Table 1:** Multivariate analysis of race/ethnicity and awareness ^*^Statistically significant

Survey question	White, n (%)	Black, n (%)	Other, n (%)	P-value
Believes sun exposure plays a role in skin cancer	191/197 (96.9)	153/213 (71.8)	54/62 (87.1)	< 0.0001^*^
Believes it is possible to get skin cancer on the eyelids	161/197 (81.7)	102/213 (47.8)	40/62 (64.5)	< 0.0001^*^
Believes it is important to protect the face from sun exposure	191/197 (96.9)	191/213 (89.7)	60/62 (96.8)	0.0088^*^
Believes it is important to protect the eyelids from sun exposure	172/197 (87.3)	155/213 (73.1)	55/62 (88.7)	0.002^*^

Preventive measures 

The use of sunscreen and wearing sunglasses were the most common preventive measures utilized by respondents: 278/484 (58%) reported wearing sunscreen, and 342/484 (71%) reported wearing sunglasses to avoid excessive sun exposure. As shown in Table 2, there were significant education-dependent behavioral differences in avoiding sunscreen noted in the analysis. Chi-squared tests of independence demonstrated that respondents with an education level of at least some college or higher were more likely than those with some high school/GED to utilize all protective measures, including sunscreen, hats, and protective clothing, seeking shade, sunglasses, and avoiding tanning booths. There was no statistically significant relationship between race, sex, or age in sun exposure prevention methods other than sunscreen use. 

**Table 2:** Multivariate analysis of the level of education and sun exposure prevention methods ^*^Statistically significant GED: General Educational Development

Survey question	Some high school/GED, n (%)	Some college/bachelor's, n (%)	Master's or doctorate, n (%)	P-value
Wear sunscreen	66/162 (40.7)	150/235 (63.8)	56/75 (74.7)	< 0.0001^*^
Wear hats with brims or protective clothing	87/162 (53.7)	146/235 (62.1)	54/75 (72)	0.0230^*^
Seek shade during peak sun hours	65/162 (40.1)	137/235 (58.3)	44/75 (58.7)	0.0008^*^
Wear sunglasses	97/162 (59.9)	186/235 (79.1)	59/75 (78.7)	< 0.0001^*^
None of the above	16/162 (9.9)	9/235 (3.8)	3/75 (4)	0.0321^*^

Seventy out of 484 (14.5%) total participants reported that they wear sunscreen on their face daily, while 188/484 (38.8%) of participants reported that they never wear sunscreen on their face. The remaining participants reported occasional sunscreen use on their face. Additionally, 115/484 (23.8%) of total participants apply sunscreen to their eyelids when they apply sunscreen to their face. Other participants responded that they do not apply to their eyelids, are unsure if they apply to them, or never apply sunscreen to their face. Further chi-squared tests of independence were used to evaluate sunscreen use and showed statistical significance among demographic groups, as shown in Tables 3-4. 

**Table 3:** Multivariate analysis of sunscreen use across age and gender ^*^Statistically significant SPF: sun protection factor

Survey question	18-24 years, n (%)	25-44 years, n (%)	45-64 years, n (%)	65+ years, n (%)	P-value	Female, n (%)	Male, n (%)	P-value
Wears sunscreen on face daily	5/33 (15.1)	30/129 (23.3)	22/173 (12.7)	13/137 (9.5)	0.0049^*^	69/321 (21.5)	1/152 (0.6)	< 0.0001^*^
Wears sunscreen with SPF > 30	20/33 (60.6)	88/129 (68.2)	93/169 (55)	51/135 (37.8)	< 0.0001^*^	189/317 (59.6)	63/150 (42)	0.0002^*^
Applies sunscreen to eyelids when applying to face	12/33 (36.4)	43/129 (33.3)	42/171 (24.6)	18/138 (13.0)	< 0.0001^*^	91/321 (28.3)	24/151 (15.9)	0.0029^*^

**Table 4:** Multivariate analysis of sunscreen use across educational level and race ^*^Statistically significant GED: General Educational Development; SPF: sun protection factor

Survey question	Some high school or GED, n (%)	Some college or bachelor's, n (%)	Master's or doctorate, n (%)	P-value	White, n (%)	Black, n (%)	Other, n (%)	P-value
Wears sunscreen on face daily	15/162 (9.3)	37/233 (15.9)	18/75 (24)	< 0.0001^*^	43/133 (33.9)	11/212 (5.2)	15/63 (23.8)	< 0.0001^*^
Wears sunscreen with SPF > 30	53/158 (33.5)	138/232 (59.5)	59/74 (79.7)	< 0.0001^*^	153/194 (78.9)	63/207 (30.4)	33/62 (53.2)	< 0.0001^*^
Applies sunscreen to eyelids when applying to face	32/162 (19.8)	58/232 (25.0)	25/75 (33.3)	< 0.0001^*^	55/194 (28.4)	37/211 (17.5)	21/63 (33.3)	< 0.0001^*^

## Discussion

Skin cancer is the most common cancer in the United States, with eyelids being particularly vulnerable due to their high exposure to sunlight. Eyelid carcinomas account for approximately 5-10% of skin cancers, and patients with long-term UV radiation exposure are known to have higher rates of developing these malignancies [1,2,3]. Initial surgical excision often fails to remove all tumor margins, necessitating more extensive and cosmetically devastating surgeries [14]. It is hypothesized that late diagnosis contributes to these poorer outcomes [14,15]. In this study, patient awareness of eyelid cancer and sun exposure was evaluated to assist primary care providers, dermatologists, and ophthalmologists in providing informed guidance on sun protection methods and eyelid cancer prevention strategies. 

Our study found that 44.3% of respondents were unaware that eyelids could be at risk for cancer, and that 42.4% are not protecting their face with sunscreen. This highlights the critical need for patient education on the risks of eyelid carcinomas, the dangers of prolonged sun exposure, and the importance of timely healthcare visits for effective prevention and treatment. To enhance patient education, it is important to identify the groups at highest risk for poor sun protection behaviors. We found that older adults, males, individuals with lower education levels, and Black patients were less likely to practice effective sun exposure prevention methods.

Future interventions may include integrating education on sun protection methods into primary and secondary school health and wellness curricula, as it increases the likelihood of this prevention strategy reaching diverse demographic populations while also promoting early utilization of sun protection and reducing long-term skin cancer risk associated with cumulative UV exposure. Additionally, ophthalmologists and primary care providers should consider incorporating skin cancer prevention counseling into standard practice for comprehensive eye exams and annual wellness visits.

Most eyelid carcinomas are diagnosed at the age of 65 years or above [3]. Older patients, defined as those aged 65 years and older in this study, reported less frequent use of daily sunscreen and were less likely to apply it to their eyelids than younger individuals. Older patients have likely maintained sun exposure habits from their youth, indicating decades of unprotected sun exposure. It is essential to educate older patients on the importance of improving sun protection strategies while simultaneously introducing these strategies to younger patients to establish good lifelong sun protection habits.

In this study, only 1/152 (0.6%) of males reported daily sunscreen use, and 24/151 (15.9%) of males used sunscreen on their eyelids at times of use. In comparison, 69/321 (21.5%) of women reported using sunscreen daily, and 91/321 (28.3%) applied it to their eyelids. This disparity in sunscreen utilization may be tied to differences in preventive care behaviors between genders. Females are more likely to self-examine their skin, which is known to correlate with earlier cancer diagnosis [16]. Additionally, they are more likely to wear protective clothing, wide-brimmed hats, and avoid outdoor activity [16]. Traditional gender roles influence health behaviors, with masculinity often linked with health-harming behaviors and femininity with health-promoting behaviors [17]. One study found that men who adhere to masculine norms are less likely to use sunscreen regularly or demonstrate knowledge about tanning dangers, suggesting that adherence to masculine norms can serve as a barrier to sun safety [18].

Based on our findings, compared with individuals with higher levels of education, those with a high school level of education or below were less likely to practice effective sun protection methods. Taken together, this evidence suggests that education level represents a socioeconomic barrier to risk reduction for eyelid cancer. Socioeconomic status has been shown to impact melanoma outcomes, with lower-income individuals often presenting later in the disease course due to limited access to preventative care and health insurance coverage [19]. Melanoma patients with Medicaid are at a significantly higher risk of advanced-stage diagnosis compared to those covered by commercial insurance [20,21]. Late diagnosis of eyelid carcinomas leads to more invasive surgical treatments, such as exenteration and enucleation [6]. 

Additionally, our study suggests that individuals with darker skin tones, who are classified higher on the Fitzpatrick scale, are less likely to use sun protection and are generally less aware of the risks of skin and eyelid cancer. These patients may face additional diagnostic challenges due to anatomical differences or variations in clinical presentation. Medical training has traditionally included fewer representations of patients with darker skin tones, with only 4-18% of images in dermatology textbooks depicting darker skin [22]. 

This study has several limitations. Firstly, patients attending an ophthalmology clinic may have greater awareness of eyelid cancer and preventive measures than the general population. In addition, the study was conducted in an ophthalmology clinic within an academic medical center, where there is typically greater emphasis on functional ophthalmology and oculoplastic care rather than cosmetic eyelid concerns. As with any survey-based study, caution is needed when interpreting self-reported data. Responses may be influenced by social desirability bias, with participants selecting answers that they perceive as more acceptable regarding sun protection behaviors. Study participants may have felt inclined to report socially desirable responses regarding sunscreen use, suggesting that actual sunscreen application may be lower than reported. This is a cross-sectional and observational study, and therefore, causal relationships cannot be established. Expanding the research to include multiple centers across urban and rural settings could better identify populations in need of targeted educational interventions. Future studies may also include a retrospective analysis of prognostic outcomes for eyelid carcinoma across different demographic groups.

## Conclusions

The findings of this study demonstrate lower awareness of eyelid cancer and less effective sun protection behaviors among groups known to be at the highest risk for eyelid carcinomas. Our study highlights the importance of patient education through public health initiatives, targeted educational efforts for higher-risk groups, and a multidisciplinary approach to prevention and counseling involving primary care providers, dermatologists, and ophthalmologists to promote earlier detection and improve outcomes for eyelid carcinoma.

## Appendices

Supplementary material

Clinic Administered Survey (prepared by authors)

Research Participant Information Sheet

Study Title: Assessing Patient Awareness of Sun Exposure and Eyelid Cancers

VCU Investigator: Nikisha Richards, MD

You are invited to participate in a research study about knowledge of sun exposure and cancers around the eye. Your participation is voluntary. In this study, you will be asked to fill out a short, 22-question survey answering questions about your knowledge of sun exposure and cancer around the eye. We will not collect any identifiable information about you if you participate in this study. Your answers will remain anonymous. You will not be paid to participate in this study. If you have any questions, concerns, or complaints about this study now or in the future, please contact: Carolyn May, maycj2@vcu.edu, 717-524-8918. If you would like a copy of the information presented to you here, please let the study team member know. 

Question 1: What is your age?

a. 18-24 years old

b. 25-44 years old

c. 45-64 years old

d. 65+ years old

Question 2: What is your sex? 

a. Male

b. Female

c. Non-binary

Question 3: What is the highest degree or level of education you have completed? 

a. Some high school

b. High school graduate or equivalent (GED)

c. Some college, no degree

d. Associate degree

e. Bachelor’s degree

f. Master’s degree

g. Doctorate degree

Question 4: Please specify your ethnicity.

a. White

b. Hispanic or Latino

c. Black or African American

d. Native American or American Indian

e. Asian/Pacific Islander

f. Other

Question 5: How often do you participate in outdoor activities (hanging out outside, going to the park, hiking, going to the beach, swimming, walking, gardening, skiing)?

a. More than once a week

b. Once a week

c. Once a month

d. Less than once a month 

Question 6: What do you think about the effects of sun exposure on the skin?

a. Good

b. Bad

c. Mild

d. I don’t know

Question 7: Do you think sun exposure plays a role in skin cancer?

a. Yes

b. No 

c. I don’t know

Question 8: How many eyelids do humans have?

a. 1

b. 2

c. 4

d. More than 4

Question 9: Do you think it is possible to get skin cancer on your eyelids?

a. Yes

b. No

c. I don’t know

Question 10: Do you think it is important to protect your face from sun exposure?

a. Yes

b. No

c. I don’t know

Question 11: Do you think it is important to protect your eyelids from sun exposure?

a. Yes

b. No

c. I don’t know

Question 12: What measures do you take to avoid excessive sun exposure? (Select all that apply)

a. Wearing sunscreen

b. Wearing hats with brims or protective clothing

c. Seeking shade during peak sun hours (10 am-4 pm)

d. Wearing sunglasses

e. Avoiding tanning booths

f. None of the above

Question 13: How often do you wear sunscreen on your face?

a. Everyday

b. Often

c. Sometimes

d. Never

Question 14: During which of the following activities would you wear sunscreen?

a. Going to the beach on a sunny day

b. Lying by the pool on a cloudy day

c. Driving in your car

d. a & b

e. a & c

f. b & c

g. All of the above

h. I do not wear sunscreen

Question 15: What SPF (sun-protection factor) do you use when you wear sunscreen?

a. SPF greater than or equal to 30

b. SPF less than 30

c. I don’t know

d. I do not wear sunscreen

Question 16: When you apply sunscreen to your face, do you apply the sunscreen on your eyelids? 

a. Yes

b. No

c. I don’t know

d. I do not wear sunscreen

Question 17: If you are outside, how often do you apply sunscreen on your face? 

a. More frequently than every 2 hours

b. Every 2 hours

c. Less frequently than every 2 hours

d. Never 

Question 18: How often do you wear sunglasses when outside?

a. Always

b. Often

c. Sometimes

d. Never

Question 19: How often do you tan either naturally or in a tanning booth/salon/spa?

a. More than once a week

b. Once a week

c. Once a month

d. Less than once a month 

Question 20: Have you ever been diagnosed with skin cancer (of any kind)?

a. Yes

b. No

c. I’m not sure

Question 21: Have you ever been diagnosed with skin cancer of the eyelid?

a. Yes

b. No

c. I’m not sure

Question 22: If yes, what type of skin cancer of the eyelid?

a. Melanoma

b. Squamous cell carcinoma

c. Basal cell carcinoma

d. Other

e. I’m not sure
